# Maximising biodiversity potential in Europe’s mines and quarries: A key role for EU Nature Restoration Regulation targets

**DOI:** 10.1007/s13280-025-02235-4

**Published:** 2025-09-03

**Authors:** Miguel Ballesteros, Klara Řehounková, Kris Decleer, Carolina Martínez-Ruiz, Josu G. Alday, Rodolfo Gentili, Alice Nunes, Pedro A. Salgueiro, Gregory Mahy, Samuel Bouchoms, Anita Kirmer, Sabine Tischew, Vicenç Carabassa, Nina Nikolic, Rob Marrs, Karel Prach

**Affiliations:** 1 Department of Botany, Faculty of Science, University of South Bohemia, Branišovská 1760, CZ-37005 České Budějovice, Czech Republic; 2 Research Institute for Nature and Forest, Herman Teirlinckgebouw, Havenlaan 88 Bus 73, 1000 Brussels, Belgium; 3 Ecology Area, Instituto Universitario de Investigación en Gestión Forestal Sostenible (iuFOR), ETSIIAA, University of Valladolid, Avda. Madrid 44, 34004 Palencia, Spain; 4 Department of Agricultural and Forest Sciences and Engineering (DCEFA), University of Lleida, Av. Alcalde Rovira Roure 191, 25198 Lleida, Spain; 5 Joint Research Unit CTFC – AGROTECNIO – CERCA, Av. Alcalde Rovira Roure 191, 25198 Lleida, Spain; 6 Department of Earth and Environmental Sciences, University of Milano-Bicocca, Piazza della Scienza 1, 20126 Milan, Italy; 7 cE3c - Centre for Ecology, Evolution and Environmental Changes & CHANGE – Global Change and Sustainability Institute, Faculdade de Ciências, Universidade de Lisboa, 1749-016 Lisbon, Portugal; 8 MED – Mediterranean Institute for Agriculture, Environment and Development & CHANGE – Global Change and Sustainability Institute, UBC – Conservation Biology Lab, Department of Biology, ECT, University of Évora, 7006-554 Évora, Portugal; 9 Gembloux Agro-Bio Tech, Biodiversity - Ecosystems - Landscapes Team, University of Liège, Passage des Déportés 2, 5030 Gembloux, Belgium; 10 Department for Nature Conservation and Landscape Planning, Anhalt University of Applied Sciences, Strenzfelder Allee 28, 06406 Bernburg, Germany; 11 CREAF, 08193 Bellaterra (Cerdanyola del Vallès), Catalonia Spain; 12 Institute for Multidisciplinary Research, University of Belgrade, Kneza Viseslava 1, 11030 Belgrade, Serbia; 13 School of Environmental Sciences, University of Liverpool, Jane Herdman Building, 4 Brownlow St, Liverpool, L3 5DA UK

**Keywords:** Biodiversity loss, Habitat restoration, Nature-based solutions, Resource extraction, Restoration guidelines, Sustainable ecosystems

## Abstract

Amid the UN Decade on Ecosystem Restoration and the EU’s Nature Restoration Regulation (NRR), which aims to restore degraded areas in the coming decades, post-mining sites must be integrated into biodiversity and ecosystem recovery strategies as key contributors. While mining, quarrying, and other extractive activities have considerable environmental impacts, they also present massive opportunities to create valuable habitats, support biodiversity, guide restoration efforts, and contribute to conservation. A strong foundation of scientific and practical knowledge is already in place, yet implementation gaps persist, and regulatory frameworks remain under-utilised for restoring these degraded areas. Under-exploited pathways exist to reconcile development needs with NRR restoration goals. To maximise the biodiversity potential of post-mining sites, we emphasise the need for: (1) Site-specific scientific assessments and long-term monitoring; (2) Practical restoration guidelines for European habitats; (3) The strategic use of restored site networks as demonstration areas; (4) Active stakeholder engagement; and (5) Supportive policies.

## Introduction

The United Nations Sustainable Development Goal 15 coupled with the ‘Decade on Ecosystem Restoration 2021–2030’ collectively emphasise the importance of advancing restoration science and the derivation of effective practices to prevent, halt, and reverse the degradation of natural habitats and biodiversity loss (UN 2019; CBD 2022). Extractive activities, including mining, quarrying, and the extraction of peat and other fossil fuels, have significant impacts on ecosystems and ecological processes, presenting urgent environmental and developmental challenges (Sonter et al. 2018; Harries et al. 2023). Addressing these challenges is essential to prevent further biodiversity loss and preserve ecosystem functions (Salgueiro et al. 2020a, b; Boldy et al. 2021).

Globally, over 66 000 km^2^ have already been altered by extractive activities, and demand for mining and quarrying products is expected to increase (Murguía et al. 2016; Tang and Werner 2023). This increasing activity largely outpaces the implementation of mitigation measures, compensatory conservation, and restoration strategies (Sonter et al. 2020; Young et al. 2022). Extractive activities result in habitat loss, pollution, soil and water degradation, species displacement, and the spread of undesirable species, which may significantly harm ecosystems and biodiversity (Murguía et al. 2016; Monty et al. 2019; Harries et al. 2023). In Europe, such activities have impacted a range of ecosystems, including sensitive habitats and species recognised as conservation priorities at regional through to international levels (e.g. Habitats Directive 92/43/EEC). As demand for raw materials continues to rise, these pressures and impacts are expected to intensify (Sonter et al. 2020; Grohol and Veeh 2023).

The EU Nature Restoration Regulation (NRR), also known as the “Nature Restoration Law” came into effect on 18 August 2024, as part of the Biodiversity Strategy for 2030 and the European Green Deal (European Parliament and Council 2024; SERE 2024a). This regulation establishes legally binding targets to achieve four objectives across the EU: (1) Restore degraded ecosystems, (2) Reverse biodiversity loss, (3) Address climate change, and (4) Enhance ecosystem resilience. It sets an ambitious goal to restore 20% of the EU’s land and sea areas, including 30% of habitat areas by 2030, with a longer-term goal of restoring all ecosystems in need by 2050. The NRR has the potential to play a crucial role in shaping the restoration of ecosystems and habitats impacted by extractive activities, but its practical implementation will require clear guidance and well-defined methodologies.

Restoration approaches range along a spectrum of activity from the least intrusive, spontaneous (passive) restoration, through active restoration, to reclamation (Chazdon et al. 2021). Passive restoration relies on ecological succession (i.e. natural regeneration) with minimal intervention (Prach et al. 2013; Pitz et al. 2018). Active restoration includes, inter alia, enhancing abiotic conditions, creating microsite heterogeneity, introducing target species propagules, controlling undesirable species, and promoting natural processes such as plant-to-plant facilitation, generally through nature-based solutions (Baasch et al. 2012; Gilardelli et al. 2016a). Reclamation uses a technically intensive approach that might involve terrain stabilisation, aesthetics, public safety, and repurposing land for productive use (Prach and Tolvanen 2016; Chazdon et al. 2021; Young et al. 2022).

However, although these various restoration approaches have been developed, there is within Europe a lack of robust guidelines and policies tailored to specific ecological contexts and (bio)geographical scales. Often, the consequence has been the transformation of sites into ecologically, low-value habitats (Castillejo and Castelló, 2010; Řehounková et al. 2023; Jurasinski et al. 2024). Sub-optimal practices such as the wholesale use of: (a) Technical restoration methods, or (b) Introducing unsuitable species (e.g. non-native, invasive, or unadapted ones) frequently fail to achieve desirable environmental restoration outcomes (Martínez-Ruiz et al. 2007; Kirmer et al. 2012; Nunes et al. 2014). In many cases, a simpler and cost-effective approach using natural regeneration would be more successful (Řehounková et al. 2012).

Irrespective, the potential of restored post-mining sites (i.e. mines and quarries) as models for developing effective ecological management and biodiversity conservation strategies remains largely unrecognised in national and EU policies (Tischew and Kirmer 2007; Hering et al. 2023; SERE 2024a).

## The potential of mining and quarrying sites to support biodiversity

Despite their well-documented damaging environmental impacts (Sonter et al. 2018; Boldy et al. 2021; Tang and Werner 2023), extractive activities can create sites with a high degree of geological and biological diversity, often standing in contrast within homogeneous and often ecologically degraded landscapes. These newly-formed sites may feature diverse geomorphological characteristics with varied topography, such as escarpments, rock walls, ledges, cavities, scree, exposed substrates, bare surfaces, and water bodies, offering potential habitats for a diverse range of aquatic and terrestrial organisms. Post-mining sites can also act as valuable surrogate habitats for rare, specialised, or threatened species, often attracting a diverse wildlife, especially in fragmented landscapes. As the substrates are often very infertile, they can serve as refuges for species that require low nutrient levels to thrive, especially in surrounding areas of high fertility, such as agricultural areas or where atmospheric eutrophication occurs. These sites can serve as temporary or permanent habitats for declining and retreating species (Kirmer et al. 2008; Salgueiro et al. 2020a; Řehounková et al. 2020; Seleck et al. 2022; Kettermann and Fartmann 2023).

The species that establish successfully on these sites can either arrive through natural colonisation by dispersal from nearby habitats, or they may be actively introduced through restoration measures. In both cases, the desirability of the established species can be assessed through a comparison with species from local reference ecosystems (Martínez-Ruiz et al. 2007; Kirmer et al. 2008; Tischew et al. 2014; Řehounková et al. 2020; Harries et al. 2023).

Post-mining sites can also provide multiple ecosystem services (Prach and Tolvanen 2016; Salgueiro et al. 2020b; Boldy et al. 2021), often simultaneously, delivering social and cultural benefits (e.g. nature-based recreation), as well as provisioning (e.g. food and timber) and regulating functions (e.g. pollination and carbon sequestration). Some post-mine sites have become so ecologically valuable that they have been designated as nature reserves, protected areas, or integrated into the EU Natura 2000 Network (European Commission 2019). However, achieving positive environmental outcomes depends not only on site-specific factors and time, but also on supportive policies, well-executed mining operations, appropriate restoration plans, and informed frameworks for the extractive sector (Prach et al. 2011; Pitz et al. 2016).

## Perspectives from researchers and practitioners in mine and quarry restoration

**Fig. 1:**
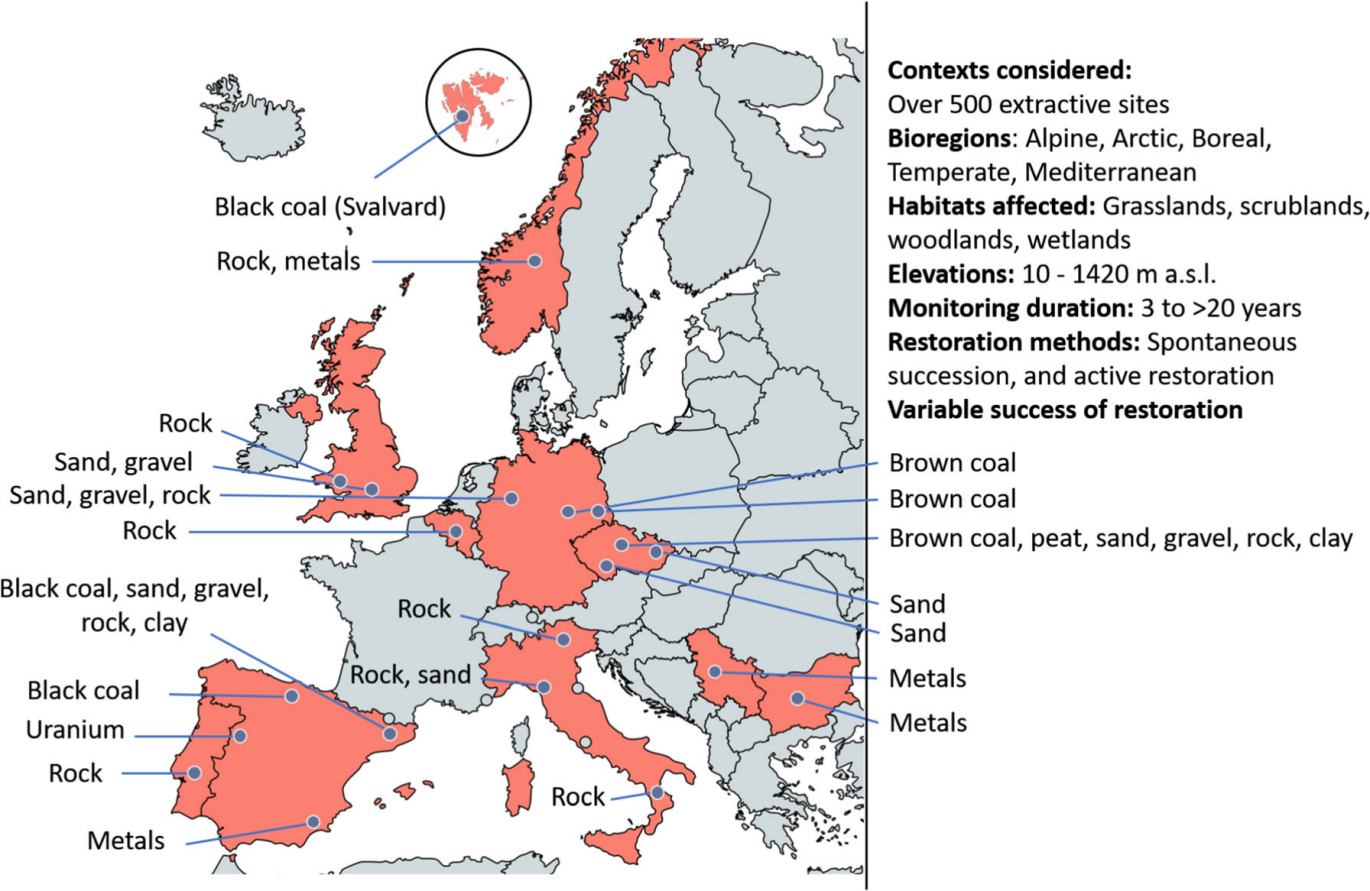
Geographic distribution of European restoration projects on mines and quarries (n > 500) discussed at the 14th European Conference on Ecological Restoration in Tartu, Estonia (2024). These sites cover the extraction of different raw materials, and they affect a range of ecosystems. The sites have undergone diverse restoration approaches, with varying levels of success. Blue circles represent more than ten post-mining sites (Belgium, Czechia, Germany, Spain, UK). Light-grey circles represent microstates (i.e. Andorra, Monaco, Vatican, San Marino, and Liechtenstein). Extracted materials include fossil fuels (coal, peat), rocks (e.g. limestone, marble, gypsum, sandstone, granite, and basalt), sand, gravel, china clay, and metal ores

In order to consolidate the views of experts, practitioners, and stakeholders on the role of ecological restoration in mines and quarries, we ran a designated session entitled “*On the Way to European Mining Restoration Guidelines*” at the 14th European Conference on Ecological Restoration in Tartu, Estonia (August 27, 2024; SERE 2024a, 2024b). This session showcased studies from over 500 extractive sites across ten European countries (Fig. 1) and the challenges and opportunities of restoring post-mining sites for biodiversity were discussed (SERE 2024b). Participants shared insights from the first steps in restoring or creating new ecosystems using a range of approaches through to ecosystem development, with some case studies describing successional change based on over 20 years of monitoring. The habitats included: grasslands, scrublands, woodlands, and wetlands. The main takeaways (summarised in Table 1) included: (1) The effectiveness of natural regeneration in restoration; (2) The importance of understanding site-specific conditions; and (3) The need for long-term monitoring to assess restoration success. It was also noted that certain post-mining sites exhibit high specificity due to the nature of the extracted material, methods of extraction, and the landscape context.

**Table 1:** Key takeaways for effective mining and quarrying restoration and improved environmental outcomes. Insights derived from the “*On the Way to European Mining Restoration Guidelines*” session at the 14th European Conference on Ecological Restoration in Tartu, Estonia (SERE 2024b). These insights are based on research and case studies shared by participants across Europe. The references provided do not endorse any practice as a universal solution but highlight the importance of considering the described takeaways

Key takeaways	Rationale and considerations	References
Understanding regional specificities	Rationale: Restoration efforts must first acknowledge the unique environmental and ecological conditions of different regions. Without this foundational knowledge, any subsequent restoration approach may be ineffective or even harmful Considerations: Addressing gaps in research and practice, especially the lack of centralised data and long-term restoration case studies in extractive sites across alpine, arctic, boreal, temperate, and Mediterranean regions, is essential to develop a critical mass of best practices to tailor restoration efforts to specific environmental contexts. The effective restoration of unique, specialised, and isolated biotic communities restricted to specific substrates (e.g. peat, serpentines, limestone, marble, gypsum, or dolomites) affected by the extractive industry requires special consideration. Effective management of alien and invasive species requires a focus on regional risks, as their impacts vary widely across habitats and can substantially hinder restoration outcomes	Gentili et al. (2011), Prach et al. (2013), Ballesteros et al. (2014), Nunes et al. (2016), Pitz et al. (2019), Jurasinski et al. (2024)
Understanding site opportunities and limitations	Rationale: Once regional factors are considered, site-specific constraints and potentials must be assessed to tailor restoration strategies appropriately Considerations: The effectiveness of restoration efforts depends largely on local site conditions, including geo-relief, soil quality, available bedding materials, macro- and micro-climatic factors, biota dispersal, and connectivity between extraction and donor sites. Therefore, identifying and addressing the key limitations, leveraging opportunities, and defining expected outcomes for specific mining and quarrying contexts is crucial. Near-natural restoration (i.e., restoration based on natural processes, either spontaneous or guided) can go beyond replicating pre-exploitation ecosystems. It may involve creating new or analogous ecosystems that, while not strictly identical to historical references, can support high biodiversity and provide essential ecosystem services. A cost–benefit analysis can help assess different restoration approaches to determine the most cost-effective strategies for the specific site conditions, ensuring efficient use of resources	Řehounková and Prach (2006), Martínez-Ruiz and Marrs (2007), Alday et al. (2011), (2012), Tischew et al. (2014), Ballesteros et al. (2014, 2017), Nikolic et al. (2016, 2018), Pitz et al. (2018), Gentili et al. (2020), Erikstad et al. (2023), Jurasinski et al. (2024)
Need to integrate ecological processes	Rationale: Recognising and incorporating natural ecological processes ensures that restoration efforts support self-sustaining ecosystems rather than relying solely on human intervention Considerations: The establishment of natural ecological processes is indicative that, beyond compositional indicators, extractive sites are being restored into self-sustainable, healthy ecosystems and able to provide services benefiting nature and people. Supporting the recovery of ecosystem services in post-mining restoration strategies may help to foster stakeholder engagement. Restoring or creating high-quality terrestrial and aquatic habitats after extraction requires clear guidelines to ensure ecological value and prevent the spread of invasive species and eutrophication	Tischew and Kirmer (2007), Gilardelli et al. (2015), Prach and Tolvanen (2016), Monty et al. (2019), Sampaio et al. (2021), Carvalho et al. (2022)
Integration of assisted and spontaneous restoration	Rationale: With an understanding of ecological processes, the balance between assisted and spontaneous restoration can be determined, optimising resource use and ecological outcomes Considerations: Assisted restoration in extractive sites remains widely used despite often performing no better than natural regeneration (i.e. spontaneous succession). Understanding the thresholds that determine whether to apply spontaneous or assisted restoration methods is crucial. While natural regeneration should be prioritised as a cost-effective approach, assisted restoration techniques can be applied in combination when spontaneous processes alone prove insufficient	Martínez-Ruiz et al. (2007), Kirmer et al. (2008), Baasch et al. (2012), Řehounková et al. (2012), Gilardelli et al. (2016a), Prach et al. (2013, 2020), Carvalho et al. (2022)
Potential of surrogate habitats	Rationale: Extractive sites can provide significant conservation value. Identifying and utilising them as surrogate habitats contributes to broader ecological connectivity and biodiversity conservation Considerations: Establishing surrogate habitats in extractive sites contributing to a Green Infrastructure network, including stepping stones and temporary areas, can provide vital support for both endangered and non-endangered species. This strategy aims to enhance regional ecological connectivity and contribute to broader conservation goals that extend beyond those of the Natura 2000 network. Many extractive sites hold great potential as habitats for low-competitive species, often specialists of nutrient-poor environments that are increasingly displaced from surrounding eutrophicated and overexploited landscapes	Salgueiro et al. (2020b), Řehounková et al. (2020), Séleck et al. (2022)
Importance of long-term monitoring	Rationale: Effective restoration requires ongoing assessment to ensure success and adaptability over time Considerations: Establishing clear, easily measurable indicators of success or failure is vital for tracking the progress of restoration projects. Long-term monitoring is essential to provide data that demonstrates the effectiveness of restoration practices and informs future restoration strategies. Evidence from multiple studies can help identify typical restoration trajectories and tailor better indicators and adaptive management measures	Alday et al. (2011), Gilardelli et al. (2016b), Nunes et al. (2016), Pitz et al. (2016), Carabassa et al. (2019), Mexia et al. (2020), Gentili et al. (2023), Paolinelli et al. (2024)
Development of practical guidelines	Rationale: After understanding all ecological and operational factors, the development of guidelines ensures that restoration strategies are actionable and replicable Considerations: A comprehensive set of mining and quarrying restoration guidelines is needed to provide practical resources for practitioners, offering actionable recommendations for restoration efforts throughout all stages of extractive operations	Ballesteros et al. (2017), Sélek et al. (2022), Řehounková et al. (2023)
Collaboration among stakeholders	Rationale: The success of restoration efforts also depends on cooperation among various stakeholders, ensuring alignment with policies and long-term commitment to restoration goals Considerations: Engaging mining and quarrying sector representatives, policymakers, environmental professionals, and local communities is crucial for the successful implementation of restoration guidelines. Collaborative efforts must help align restoration practices with national and EU-level environmental policies	Prach et al. (2011), Pitz et al. (2016), SER (2024b)

While substantial knowledge on ecological restoration exists, it remains fragmented and under-utilised, limiting its broader application by both extractive site managers and conservation bodies. The session emphasised the need for a set of comprehensive restoration guidelines and fostered dialogue on their development to provide practical resources for practitioners. These guidelines must provide actionable recommendations for management and restoration throughout all stages of extractive operations, prioritising biodiversity conservation goals before, during, and after the extractive activities. The importance of collaboration among stakeholders, including representatives of the mining and quarrying sectors, practitioners, policymakers, local communities, and the general public, to implement these guidelines was emphasised. A key output from these discussions was the need to establish a consolidated network of restored post-mining sites to demonstrate their value for nature conservation and guide restoration efforts, highlighting its potential benefits. Further efforts are necessary to identify key actors, challenges, and good practice. We are currently working towards this goal as an informal group under the aegis of the Society for Ecological Restoration—Europe (SERE).

## Advancing restoration: Barriers and pathways

Barriers that hinder the effective implementation of restoration measures in post-mining sites across Europe (Table 1) include overly generic legal frameworks with limited scope and ambition for regulating restoration targets in the extractive sector. Non-compliance with the Extractive Industry Waste Management Directive (Directive 2006/21/EC) persists in some regions, while national and regional authorities often provide insufficient monitoring and oversight of restoration plans. While Directive 2003/35/EC promotes public participation in environmental decision-making, incentives for stakeholders and practitioners to engage in restoration efforts are insufficient. Additionally, evidence-based restoration planning is limited by a lack of knowledge on the types of recovery patterns that occur in different (bio)geographical regions. This arises from (a) A lack of long-term monitoring and (b) Site-specific scientific assessments, and even where these exist, Knowledge Exchange to managers in charge of implementation is lacking. Practical, tailored restoration guidance is needed to help stakeholders address context-specific constraints and identify the most suitable restoration targets, measures, and alternatives. Finally, greater support is required to establish a demonstration network of restored sites to show good practice, enhance environmental protection strategies, and add value to the EU Green Infrastructure Strategy (European Commission 2024).

**Fig. 2:**
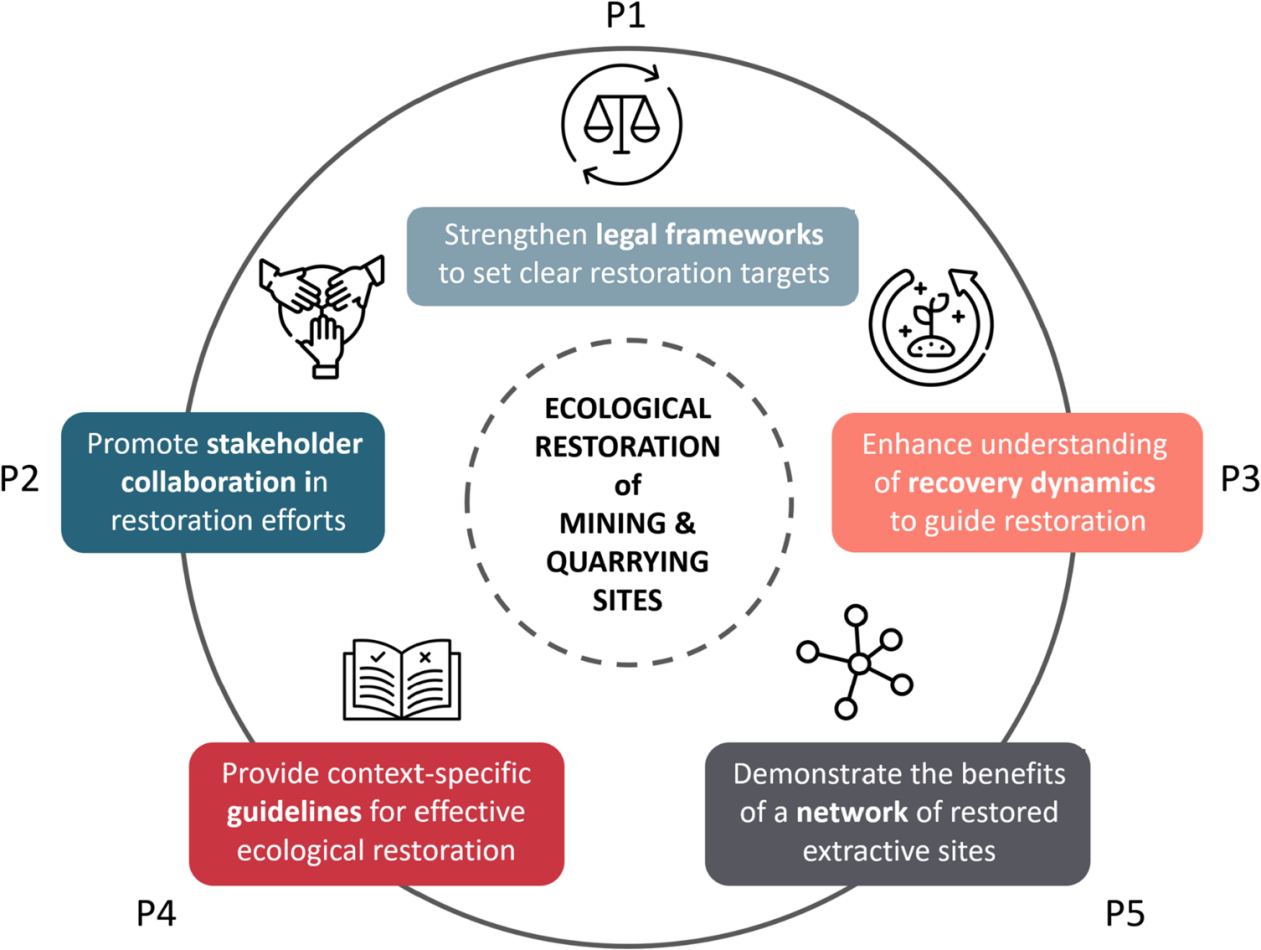
A Framework for advancing ecological restoration of mines and quarries. Key strategies to improve restoration outcomes include regulatory support, stakeholder collaboration, integration of scientific and practical knowledge, tailored practical guidelines, and the collective benefits of an inter-connected network of restored sites. The pathways (P1–P5) are further detailed in Table 2, where they are linked to specific barriers and goals aimed at maximising biodiversity potential in extractive sites. Figure icons: https://www.flaticon.com

Addressing these barriers reveals synergistic pathways to enhance the ecological and conservation potential of post-mining sites (Fig. 2; Table 2). Figure 2 presents a framework that integrates socio-economic and ecological aspects, with additional explanation in Table 2. This approach involves:

- Strengthening legal and regulatory measures for nature protection, impact assessment, and habitat restoration;
- Engaging stakeholders and practitioners in implementation;
- Advancing scientific assessment to guide restoration efforts;
- Developing tools for accessing and disseminating site-specific environmental information, tailoring guidelines to identify solutions for specific limitations;
- Demonstrating the potential of restored post-mining sites for habitat creation and enhancing connectivity in fragmented landscapes.

**Table 2:** Barriers and pathways for maximising biodiversity potential in Europe’s extractive sites. Reconciling development and conservation presents significant barriers (B1–B5), which can be partly addressed by targeting various pathways (P1–P5, as illustrated in Fig. 2). These pathways aim to achieve environmental goals that maximise biodiversity support in extractive sites, contributing to the Nature Restoration Regulation targets. Goals, general recommendations, and actors are outlined. **Abbreviations for actors:** *EU Inst. *(EU Institutions), *Nat. Gov.* (National Governments), *Env. Agencies* (Environmental Agencies), *Ext. Comp.* (Extractive Companies), *Res. Inst.* (Research Institutions, including academia and R&D), *Cons. Orgs.* (Conservation Organisations), *Loc. Comm.* (Local Communities), *NGOs* (Non-Governmental Organisations), *Gen. Pub.* (General Public)

Barriers (B) & Pathways (P)	Goals	Recommendations	Actors (Main roles are indicative, not exclusive)
B1. Limited legal frameworks → P1. Strengthen legal and regulatory frameworks	Develop and enforce strong regulatory frameworks to protect nature and biodiversity Align national legislation with EU policies to support near-natural restoration Ensure robust assessment regulations to evaluate impact, planning, mitigation, and enforceable targets Develop financial incentives for stakeholders to invest in ecological restoration	Engage legal and environmental experts to refine, update, and ensure effective implementation of regulatory frameworks Develop guidance programs for implementing regulations, outlining steps, timelines, responsibilities, and reporting mechanisms Require mining operations to align with national conservation policies and nature protection networks in permitting policies Prohibit extractive activities in irreplaceable, high-value, or highly sensitive habitats Promote transnational ecological restoration efforts to strengthen environmental connectivity across borders Include integrated spatial planning to coordinate land-use and restoration efforts Develop funding mechanisms to support high-quality restoration and engage all stakeholders for long-term sustainability	*EU Inst.* (develop/align policies, engage experts) *Nat. Gov.* (develop/enforce regulations, engage experts) *Env. Agencies* (enforce regulations, compliance, assessment) *Res. Inst.* (guidance) *Ext. Comp.* (implementation, compliance)
B2. Poor implementation of restoration practices → P2. Foster stakeholder engagement and collaboration	Provide structured guidance to integrate restoration planning into mining and quarrying operations Ensure ongoing project support to facilitate compliance and implementation Establish monitoring frameworks to assess restoration progress and long-term success Ensure restoration targets are met during and beyond the extractive lifecycle	Strengthen collaboration among industry, government, academia, NGOs, practitioners and local communities to enhance transparency, trust, and compliance with regulations and best practices Facilitate knowledge transfer and address regulatory, technical, and cultural barriers to ensure the consistent application of effective restoration practices Ensure continuous evaluation and updates based on restoration outcomes, incorporating new data and methodological improvements Provide training and capacity-building to stakeholders to implement evidence-based restoration guidelines Engage extractive companies to integrate ecological and social benefits into cost–benefit assessments to optimise restoration, emphasising nature-based solutions Enforce early and progressive restoration throughout the extractive site lifecycle to prevent ecosystem degradation from cumulative impacts and optimise restoration efforts Ensure long-term post-mining and quarrying stewardship with monitoring, care, and adaptive management to sustain ecological recovery Raise awareness among local inhabitants about restoration efforts to foster community engagement and support	*EU Inst., Nat. Gov., Cons. Orgs., NGOs* (financial incentives) *Ext. Comp.* (implementation) *Env. Agencies *(guidance, compliance) *Res. Inst.* (scientific assessment, guidance) *Cons. Orgs., Loc. Comm., NGOs* & *Res. Inst.* (knowledge transfer, collaboration)
B3. Insufficient ecological knowledge and data gaps → P3. Enhance understanding of recovery dynamics to guide restoration	Use site-specific and long-term environmental data to refine restoration strategies Develop robust metrics and monitoring tools to assess restoration effectiveness Identify large-scale recovery trends and site potentials to optimise restoration approaches across different (bio-)geographical scales Facilitate knowledge transfer and stakeholder engagement	Conduct pilot studies in various regions to evaluate restoration approaches, refining guidelines based on real-world data Establish key metrics and standard monitoring techniques to adequately characterise reference communities and track restoration progress Identify the conditions under which natural regeneration can effectively meet restoration targets and prioritise its use where feasible Identify critical thresholds for species that either benefit from or are negatively affected by habitat disturbances, requiring targeted interventions (e.g. grazing, thinning, or trampling) Incorporate climate-adaptive restoration strategies, selecting plant communities and restoration designs that enhance resilience to environmental changes Implement adaptive management strategies that adjust restoration plans based on continuous monitoring and emerging ecological data Make monitoring data available to relevant stakeholders to support conservation planning and decision-making, (e.g. protected habitats, species, and red-list species) Improve the knowledge transfer from research centres to restoration practitioners Engage the public in restoration efforts through education, outreach programs, and participation	*Res. Inst.* (scientific assessment & guidance) *Env. Agencies* (monitoring, assessment) *NGOs* & *Cons. Orgs.* (knowledge transfer, outreach) *Gen. Pub.* (education & collaboration)
B4. Insufficient guidance tailored to specific contexts → P4. Provide practical, context-specific guidelines	Identify restoration measures suited to specific mining and quarrying conditions, determining where which, and how to implement them Develop and implement practical guidelines tailored to specific mining and quarrying contexts to optimise restoration outcomes Develop and implement restoration plans that consider regional biodiversity priorities and local environmental constraints Centralise knowledge to support collaboration and decision-making, including case studies, guidelines, best practices, and regulations	Develop tailored restoration guidelines based on ecological conditions, local context, and extractive practices to optimise planning, material sourcing (biotic and abiotic), and long-term sustainability Use locally adapted solutions and a case-by-case approach to address the unique environmental, social, and policy conditions of each location Set clear, measurable restoration objectives, including area shares and success indicators Incorporate landscape-scale restoration planning that considers both the extractive site and its surrounding ecosystems, recognising cumulative impacts from multiple extractive projects Guide habitat restoration plans to identify and include the re-establishment of critical habitats for sensitive species before, during, and after extractive activities Prioritise near-natural restoration and, where applicable, create new or analogous ecosystems enhancing biodiversity and ecosystem services, potentially exceeding pre-exploitation conditions Establish a centralised platform dedicated to the restoration of extractive areas to document best practices, in situ case studies, and guide future projects in similar contexts	*Res. Inst.* (guidance, knowledge transfer, outreach) *Ext. Comp.* (implementation, compliance) *Env. Agencies, Ext. Comp.* & *Cons. Orgs.* (centralise knowledge, collaboration) *Nat. Gov., Env. Agencies *& *Cons. Orgs.* (ensure biodiversity priorities)
B5. Underutilisation of restored sites for conservation → P5. Demonstrate the benefits of a network of restored extractive sites	Showcase best restoration practices through in situ case studies Demonstrate successful habitat creation that supports native and local biodiversity, while benefiting local communities Strengthen the connectivity of restored areas within broader conservation networks, linking them with existing (semi-)natural sites Prove the conservation value of restored sites beyond designated areas (e.g. Natura 2000, protected areas) and their role in enhancing European Green Infrastructure	Establish temporary habitat management and alternative sites to protect species throughout the extractive operation lifecycle Conduct surveys of sensitive species, avoid sensitive locations and times, and suspend harmful activities during migration, nesting, or flowering seasons Use or prepare areas to maximise habitat establishment with biodiversity-friendly methods (e.g. avoid nutrient-rich materials that promote undesirable species, and preserve raw substrates for specialised flora and fauna) Promote landscape heterogeneity by incorporating various successional stages, semi-open habitats, nutrient-poor conditions, and dynamic mosaics Establish minimum habitat area requirements to support viable species populations and prevent ecological fragmentation Encourage proactive habitat restoration in surrounding areas to buffer the impacts of extractive activities on local biodiversity Integrate restored extractive sites into landscape-scale ecological networks by promoting corridors that enhance connectivity and facilitate species dispersal, while mitigating associated risks Promote nature-based solutions to support biodiversity conservation	*Ext. Comp.* (implement projects) *Res. Inst.* (scientific validation, data collection) *Ext. Comp., Env. Agencies, Res. Inst., Cons. Orgs.* & *NGOs* (showcase best practices, collaboration) *Nat. Gov., Env. Agencies* & *EU Inst.* (support conservation networks) *Loc. Comm.* (collaboration)

Key actors in this process include policymakers at the EU and national levels, academia providing necessary tools and knowledge, and stakeholders and practitioners offering practical expertise and implementation support.

Our aims are to support biodiversity and address the challenges of post-mining restoration (Fig. 2, Table 2) while aligning with the NRR and its ambitious targets. Restoring mining and quarrying sites can contribute to the NRR’s goal of restoring degraded ecosystems within the EU, improving habitat conditions and reversing biodiversity loss. Targeted actions can create diverse habitats in post-mining sites, e.g. forests, scrublands, grasslands, and wetlands. All of these ecosystem types will contribute to ecosystem services such as pollinator recovery, water regulation, carbon sequestration, and erosion control. At the same time, these newly-created sites will help to reconnect ecological networks and landscapes within and beyond mining areas. Our framework advocates for the integration of clear, well-defined restoration targets into national policies through expert knowledge, stakeholder engagement, financial incentives, and structured project support, in line with the law’s mandates for National Restoration Plans and funding mobilisation. Member States are required to submit national restoration plans to the European Commission, detailing measures, monitoring, and reporting mechanisms, while aligning with key EU policies like climate change, adaptation, land degradation neutrality, and disaster prevention to prioritise restoration efforts.

Clearly, ecological knowledge is central to this strategy and accordingly we emphasise the importance of investing in site-specific data collection, long-term monitoring, and adaptive management to support the NRR’s emphasis on evidence-based decision-making and continuous assessment. However, as the implementation of sub-aspects of the NRR depends on the adaptation of individual Member States’ policies, it is inevitable that national legislation on the restoration of extractive areas will vary between States. It is, therefore, crucial to establish an ambitious baseline for restoration goals. Clear, unified targets should be set to ensure all Member States commit to advancing environmental progress within their respective policies. To ensure practical application, we encourage the development of tailored guidelines that bridge broad restoration targets with on-the-ground implementation. Lastly, by demonstrating the conservation value of restored post-mining sites, we reinforce the NRR’s objective of enhancing biodiversity beyond protected areas and strengthening ecological resilience across broader landscapes. This framework places post-mining restoration as a key contributor to the EU’s wider environmental restoration strategy.

## Applying EU regulations to promote environmentally sustainable extractive practices

The EU has provided overarching legislation and policies to help synergise existing and new regulations to maximise environmental benefits. However, this top-down legislative approach requires the engagement of informed policymakers and professionals to ensure high-quality, on-the-ground regional implementation (Table 2, B1-P1). This collaboration is crucial for developing a legal framework that protects nature while supporting strategic planning in the extractive industry. Part of the key regulatory Framework encompasses: (1) Strategic Environmental Assessments (Directive 2001/52/EC); (2) Environmental Impact Assessments (Directive 2014/52/EC); (3) Article 6 of the Habitats Directive which permits certain procedures under; (4) the Directive on the Management of Waste from Extractive Industries (2006/21/EC; (5) the Water Framework Directive (2000/60/EC); and (6) the Groundwater Directive (2006/118/EC). Additional relevant regulations may apply depending on the specific context.

The recently-adopted NRR offers additional legal powers which have the potential to transform change, making extractive activities less damaging and more supportive of biodiversity and ecosystem services. The recent European Commission guidance (European Commission 2019, 2021) can help expedite progress in this direction. However, to implement this across Europe will require engaging legal and jurisprudence experts, first, to clarify those procedures which are legally binding, and second to ensure their effective implementation (SERE 2024a). As mentioned previously, to do this requires the active involvement of stakeholders and environmental practitioners, alongside guidance from academia and R&D institutions, including governments, industry, consulting firms, NGOs, and local communities (Table 2, B2-P2). Unfortunately, without the involvement of all relevant actors, there is a large gap between EU environmental policies with its ambitious targets and the practical means to achieve them (Hering et al. 2023; SERE 2024a).

While part of the extractive industry has welcomed the EU Biodiversity Strategy for 2030 and views the NRR intention positively (e.g. AE-UEPG et al. 2023), concerns persist about the sector’s limited involvement in developing the NRR and the potential negative impacts of its regulatory power on its profit margins. The sector fears that overly-ambitious goals and retroactive measures could disincentivise investment and undermine its autonomy through EU-level decision-making. While NRR’s overarching legal framework has only recently come into force, we argue that there are still underdeveloped pathways offering an opportunity to reconcile development and conservation goals, helping to improve the transition from business-as-usual extractive approaches to more sustainable and nature-friendly practices.

## Bridging science and practice for effective ecological restoration

**Fig. 3:**
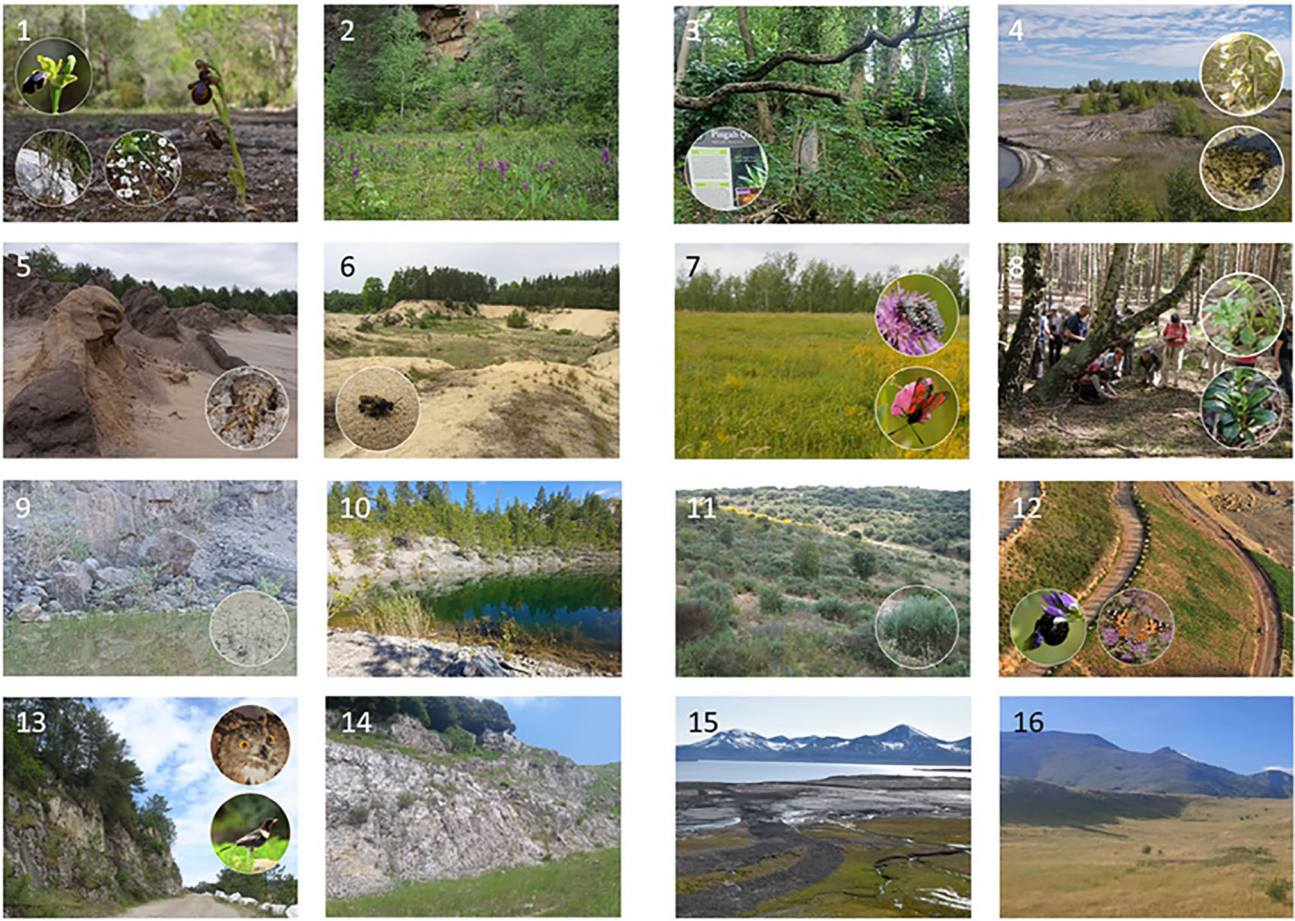
Mining and quarrying sites as surrogate habitats supporting biodiversity and ecosystem services from local to regional scales. Locally, aside from the restored middle and late successional stages that provide stable habitats for long-term ecological processes, early successional habitats (e.g. semi-open areas, temporary ponds, shallow, and nutrient-poor substrates) serve as refuges for low-competitive, specialist, and endangered species. Regionally, restored areas and diverse landforms contribute to landscape heterogeneity, habitat creation, and ecosystem services (see numbered images below). **Numbered images**: Examples of biodiversity support in post-mining sites across the EU (with Norway and the UK included for demonstration), illustrating various mining and quarrying types, habitats, and restoration methods. Images show positive environmental outcomes, not asserting them as best practices, but highlighting the potential of post-mining areas to support biodiversity through natural regeneration or assisted restoration. (1) **Limestone quarry—Portugal (Mediterranean)** Shallow, calcareous substrates support rare orchids, daffodils, and other rare endemic plants restricted to valuable open dry grassland and scrub formations. Method: Spontaneous colonisation of sites subject to assisted restoration. Photo: Pedro Salgueiro, Alice Nunes, and Cláudia Mendes. (2) **Acidic stone quarry—Czech Republic (Temperate-Continental)** Shallow, nutrient-poor soils on the quarry floor create conditions that limit vegetation overgrowth while supporting rare and endangered plant species, such as the vulnerable *Dactylorhiza majalis* orchid (Czech Red List 2017), shown in the image. Method: Natural regeneration. Photo: Karel Prach. (3) **Limestone quarry—Wales, UK (Temperate-Atlantic)** Converted into an urban biodiversity reserve, abandoned quarries enhance green infrastructure by connecting fragmented landscapes and providing recreational services. Method: Natural regeneration. Photo: Miguel Ballesteros. (4) **Brown-coal mining area—Mücheln, Germany (Temperate-Continental)** Once a brown-coal mining site, the site is now part of the Geiseltal Nature Reserve (core zone, no public access). After over 25 years of natural regeneration, a biotope mosaic of bare soil, dry grassland, reeds, shrubs, and trees has developed. Method: Natural regeneration. Photo: Anita Kirmer. (5) **Brown-coal mining area—Plessa-Grünewalde, Lusatia, Germany (Temperate-Continental)** Surface-mining of lignite creates dynamic habitat mosaics. While more hospitable areas quickly develop into woodlands, inhospitable substrates remain open for extended periods, supporting specialised species, including ground-dwelling insects. The persistence of these open spaces is essential for maintaining biodiversity in post-mining landscapes. Method: Natural regeneration. Photo: Anita Kirmer and Christian Hildmann. (6) **Sandpits—Czech Republic (Temperate-Continental)** Open grasslands formed in sandpits support endangered flora and entomofauna, including sand-nesting wild bees and wasps. Moderate trampling creates gaps, preventing the overgrowth of competitive plant species. Method: Natural regeneration. Photo: Jiří Řehounek. (7) **Brown-coal mining site—Roßbach, Geiseltal, Saxony-Anhalt, Germany (Temperate-Continental)**. A restored slope consisting of loess material, with green hay transferred from a nature conservation area (harvested in September 2000). After 19 years, the vegetation is dominated by target grassland species offering foraging ground for pollinators. Method: Assisted restoration. Photo: Anita Kirmer. (8) **Brown-coal mining area—Kromlau, Muskau Arch, Lusatia, Germany (Temperate-Continental)**. A circa 100-year-old steppe pine forest (Pyrolo-Pinetum) developed from pine afforestation, now hosting spontaneously emerging rare Pyrolaceae and Lycopodiaceae. Method: Assisted restoration and spontaneous colonisation. Photo: Anita Kirmer. (9) **Limestone quarry—England, UK (Temperate-Atlantic)**. Early-successional habitats and temporary ponds provide critical breeding sites for amphibians and other fauna in altered landscapes. Amphibians are among the most rapidly declining groups worldwide. Method: Natural regeneration. Photo: Miguel Ballesteros. (10) **Oil shale mine—Estonia (Boreal)**. Oil shale mines were afforested and water bodies created, but non-restored open areas have been spontaneously colonised by diverse flora, including orchids and Pyrolaceae species, highlighting the potential for rare biodiversity. Method: Natural regeneration, in some areas. Photo: Karel Prach. (11) **Uranium Mining—Western Spain (Mediterranean)**. Post-mining sites with assisted restoration using arkoses over uranium waste support endemic shrubby species, such as *Cytisus multiflorus*, a legume endemic to the northwest Iberian Peninsula in Dehesa formations. Method: Assisted restoration. Photo: Carolina Martínez-Ruiz. (12) **Limestone quarry—Portugal (Mediterranean)**. An initial recovery strategy favouring ruderal, flowering herbaceous layers supports insect pollinators, especially wild bees and flies, by providing essential foraging resources. Exposed soil patches within the quarry also offer ideal nesting sites for ground-nesting bee species. Method: Assisted restoration. Photo: Pedro Salgueiro and Cláudia Mendes. (13) **Limestone quarry—Portugal (Mediterranean)** Steep cliffs, walls, ledges, and rocky crevices, mimic natural conditions for breeding and wintering species such as the eagle-owl, ring ouzel, and black redstart. The effectiveness of quarry landforms as surrogate habitats is enhanced when surrounding land use is unsuitable for these species, such as in homogeneous agricultural landscapes. Method: Spontaneous colonisation of sites subject to assisted restoration. Photo: Pedro Salgueiro and Cláudia Mendes. (14) **Limestone quarry—Central Alps, Italy (Alpine)** Quarry walls support rare chasmophytic flora, while quarry floors and ledges harbour scattered patches of dry grassland communities that attract rare butterflies and birds. Method: Natural regeneration. Photo: Rodolfo Gentili. (15) **Coal mining site—Svalbard, Norway (Arctic)** Restoration of geo-biodiversity and natural processes like glacial, slope, and fluvial dynamics involves removing roads, housing, and landfills. Revegetation, though a priority, is slow in Arctic conditions and will take decades to cover bare surfaces. Method: Natural regeneration. Photo: Dagmar Hagen. (16) **Open-cast black-coal mining site—Northern Spain (Mediterranean)**. Transformed into a managed grassland for extensive sheep grazing, the site restores habitats for local grassland species, including birds, insects, and semi-arid flora. Method: Assisted restoration. Photo: Josu G. Alday.

Across Europe, the legacy of extractive sites illustrates both the limitations of environmental recovery and the varying success of restoration strategies, with a few examples shown in Fig. 3. However, this information is often difficult or impossible to access because it is scattered, decentralised, confidential, or unassessed (Table 2, B3-P3). Scientific assessment and monitoring of post-mining sites play a crucial role in identifying both failures and successes in restoration, guiding good practice across similar sites and regions, and providing a realistic reference for the timescales required (Prach et al. 2013; Harries et al. 2023). Such evaluation allows ineffective measures to be discarded and hence improve the overall efficiency of restoration investments.

Although some knowledge has been partly integrated into policy and restoration standards (European Commission 2019, 2021; Young et al. 2022), the principles are broad and there is limited transferability, which together compromise restoration success (Hering et al. 2023; Řehounková et al. 2023). Despite a strong ecological foundation and available technical capacity, context-specific guidelines for ecological restoration in mining and quarrying remain scarce, inaccessible, and underdeveloped (Table 2, B4-P4). Technical literature predominantly focuses on engineering aspects, often neglecting practical considerations essential for effective environmental recovery. The lack of guidance from environmental experts in the initial permission and planning stages of extractive activities has often resulted in missed opportunities for biodiversity conservation and the integration of nature-based solutions in restoration projects. Even when such knowledge is available, companies often fail to integrate it effectively and frequently exclude environmental experts.

In many cases, scientific knowledge on the ecological restoration of post-mining sites is advanced and readily applicable, yet engagement and knowledge exchange with practitioners remain limited. Some practitioners involved in reclamation even actively reject scientific recommendations, prioritising short-term financial gains over ecological concerns. Others are, either unfamiliar with effective ecological restoration methods or rely on long-established but often less-effective practices (e.g. allochthonous nutrient-rich materials or species-poor planting and seeding; Martínez-Ruiz et al. 2007; Castillejo and Castelló, 2010; Prach et al. 2011; Kirmer et al. 2012; Nunes et al. 2014; Jurasinski et al. 2024), and are resistant to different approaches. Aside from the specific technical aspects of each mining context (e.g. site stabilisation, propagule introduction methods, and management), we provide recommendations to better integrate conservation principles, improve ecological outcomes, and support long-term sustainability (see Table 2). Whilst not comprehensive or definitive, these offer important considerations for improving restoration outcomes. The absence of practical, region-specific guidelines based on long-term case studies tailored to mining and quarrying contexts remains a major barrier to effective implementation of ecological restoration (Řehounková et al. 2023). Future guidelines should provide clear, context-specific recommendations on where and how to implement restoration measures for long-term sustainability.

## Using post-mining sites for synergies in restoration and conservation

We suggest establishing a network of ecologically valuable, post-mining sites where target biological components are scientifically assessed and monitored, would exemplify good practice. Demonstrable improvements in biodiversity and conservation would not only be positive in themselves, but also serve as in situ demonstrations of effective restoration, guiding efforts in similar contexts (Table 2, B5-P5; Fig. 3). Additionally, restored post-mining sites can be integrated into surrounding (semi-)natural areas or function as stepping stones within fragmented landscapes, strengthening ecological connectivity and complementing the role of protected areas. In some cases, well-designed mining and quarrying restoration projects can support the establishment of habitat types and species protected under the Habitats (92/43/EEC) and Birds Directives (79/409/EEC), contributing to national and international efforts to address the biodiversity crisis.

Environmental and ecological guidance can play a key role in designing exploitation plans, implementing effective management practices during extractive operations, and addressing restoration after site abandonment. These restored sites could contribute to nature recovery, while complementing Green Infrastructure and the role of areas primarily designated for the conservation of well-preserved habitats, such as Natura 2000 in Europe. This approach also offers transnational benefits by promoting cross-border ecological connectivity and shared conservation efforts across the EU and beyond.

Restoration efforts, when effective, should build upon strong conservation strategies, reinforcing rather than replacing them (i.e. nature protection preceding restoration). Extractive activities often result in habitat destruction, pollution, soil and water degradation, species displacement, or the spread of invasive or undesirable species (Sonter et al. 2018; Boldy et al. 2021). Therefore, even though a positive end-result for conservation can be achieved through restoration this should not be used to justify new mining and quarrying activities. Post-mining site restoration, if lacking ecological assessments and demonstrated substantial ecological value, should not be used to artificially inflate outputs to meet the NRR’s ecosystem restoration targets for 2030 and beyond. Instead, we argue that well-guided mining and quarrying projects, avoiding environmentally or culturally valuable sites, can create new sites of high ecological value, offering restoration and conservation opportunities—particularly when integrated into a strategic, large-scale network. Before approving new extractive activities, a net impact assessment should weigh the site’s natural and cultural value against commitments to achieving ambitious and well-justified, ecological outcomes throughout the entire lifespan of the extractive operation. Despite the scope of opportunities they offer, post-mining sites remain under-utilised in advancing broader conservation goals. However, with proper scientific validation, recognition, and realisation of their full potential, post-mining sites could play a critical role in halting biodiversity loss and contributing to the long-term ecological recovery of degraded areas in Europe (UN 2019; European Parliament and Council 2024; SERE 2024a).

## Data Availability

Data sharing is not applicable to this article as it does not involve the generation of new data.
